# Genetic diversity and population structure of *Bellamya purificata* in Guangxi

**DOI:** 10.1371/journal.pone.0305197

**Published:** 2024-06-25

**Authors:** Chang Yuan, Zhe Li, Kangqi Zhou, Xianhui Pan, Yusen Li, Caiqun Zhang, Yong Lin, Jinxia Peng, Zhong Chen, Junqi Qin, Xuesong Du, Yin Huang, Shengjie Zhang, Xiaokai Wei, Pingping He, Pinyuan Wei

**Affiliations:** 1 Guangxi Key Laboratory for Aquatic Genetic Breeding and Healthy Aquaculture, Guangxi Academy of Fishery Sciences, Nanning, Guangxi, China; 2 Fisheries College, Hunan Agricultural University, Changsha, Hunan, China; 3 Guangxi University, College of Animal Science and Technology, Nanning, China; 4 Southwest University, Chongqing, China; CIFRI: Central Inland Fisheries Research Institute, INDIA

## Abstract

*Bellamya purificata* is an important medicinal value and economically farmed species in China. However, because little is known about the genetic characteristics of this species, the utilization of high-quality germplasm resources is hindered. The study examined the genetic differentiation between, and the structure of 12 *B*. *purificata* populations in Guangxi using 7 microsatellite DNA markers. High genetic diversity occurred in each population, with mean observed heterozygosity 0.655 and a mean expected heterozygosity 0.832. Analysis of molecular variance reveals genetic diversity to be greater within (95.2%) than among populations (4.8%). Genetic differentiation between populations is weak (*F*st = 0.048, *P* < 0.001), with mixing of genetic clusters prevalent at the level of the individual. Genetic flow exists between populations (*N*m = 3.084–11.778), with Longshui and Guilin populations exchanging frequently. A Mantel test reveals a low correlation between geographic and genetic distances (r = 0.2482, *P* < 0.071), suggesting that dispersal between neighboring populations facilitates population exchange. No significant heterozygosity excess was observed for any population (*P* > 0.05), indicating a lack of recent genetic bottlenecks. The results provide important genetic information for *B*. *purificata*, and data for potential germplasm discovery and aquaculture development.

## 1. Introduction

The freshwater field snail *Bellamya purificata* (Viviparidae) is an economically important species unique to China that is widely distributed in rice fields, rivers, and swampy areas [1]. This snail has high medicinal, ecological, and food values and is popular among both consumers and farmers alike, with annual consumption exceeding 1 × 10^6^ tons [1]. While research on these snails has examined their culture, ecology, and nutritional value, little research has been performed on their genetic characteristics [2–4].

Microsatellite markers (sample sequence repeats, SSR) are informative, reproducible, and affordable, and they have been widely used to study invertebrate population genetic diversity [5, 6]. For example, for the scallop *Patinopecten yessoensis* [7], oyster *Magallana sikamea* (as *Crassostrea sikamea*) [8], and clam *Meretrix meretrix* [9]. While genetic diversity in *B*. *purificata* has been reported [10], the genetic composition of snails in Guangxi, where the “Liuzhou river snails rice noodle” dish originated, has not.

In terms of water resources, the Guangxi Zhuang Autonomous Region is one of the wealthiest provinces in China, with more than 900 rivers contributing to a catchment area exceeding 50 square kilometers, including the Xijiang, Yangtze, and coastal river systems [11]. It is important to investigate the population genetic diversity of *B*. *purificata* in this aquatic system to better appreciate its germplasm resources. Accordingly, the study report the genetic structure of *B*. *purificata* in Guangxi using seven microsatellite markers on snails collected from 12 geographically separated populations.

## 2. Materials and methods

### 2.1 Sample collection and DNA extraction

**Fig 1:**
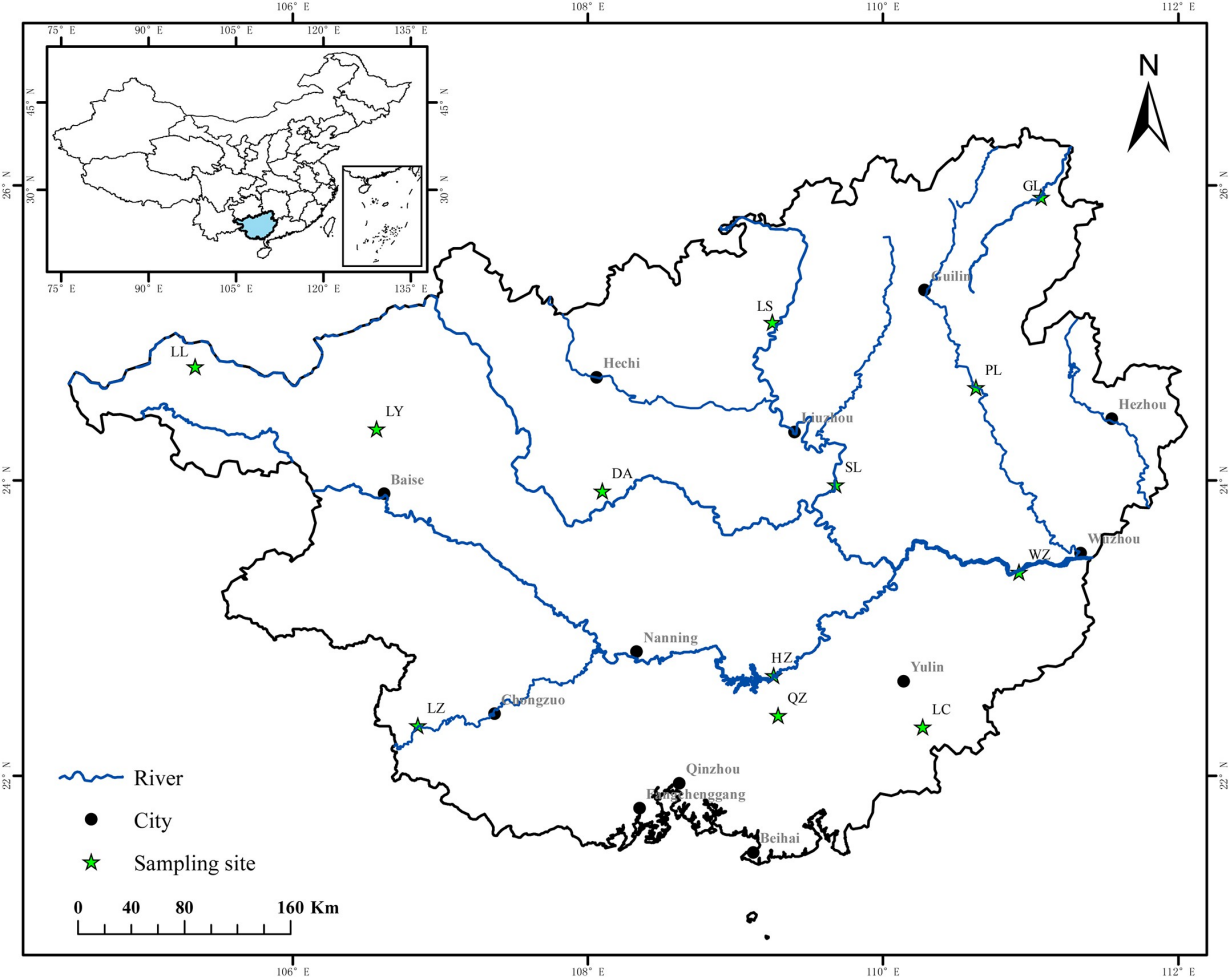
Distribution of *Bellamya purificata* sampling (yellow pentagrams) in Guangxi.

A total of 360 *B*. *purificata* individuals were collected, 30 each from 12 populations (Longlin (LL), Hezhou (HZ), Pingle (PL), Wuzhou (WZ), Longzhou (LZ), Luchuan (LC), Shilong (SL), Du’an (DA), Qinzhou (QZ), Longshui (LS), Guilin (GL), and Lingyun (LY)), throughout the Guangxi Zhuang Autonomous Region (Fig 1). All snails were placed into ice-filled boxes after collection and transported to the Guangxi Academy of Fishery Sciences, Nanning, China. The snail samples were cleaned on the shell surface and anaesthetised to death using 150 μl/g diethyl ether anaesthetic, then the shells were broken and the muscle tissue of each sample was extracted and stored in anhydrous ethanol. Muscle samples from each snail were then dissected, and genomic DNA was extracted using a Genomic DNA Extraction Kit (TIANGEN, DP324, China) following manufacturer protocols. Genomic DNA was separated on a 1.5% agarose (Biowest) gel, detected with SYSTEM GelDoc XR + IMAGE LAB (Bio-Rad), and stored at −20°C until use.

### 2.2 PCR amplification and microsatellite genotyping

Seven primer pairs were used in this study, according to Jin *et al*. and Gu *et al*. [10, 12]. M13-tailed primers (5’ TGTAAAACGACGGCCAGT 3’) were added to the 5’ direction of the upstream primer of each primer pair to synthesize splice sequences with different fluorescent groups (Table 1). Polymerase chain reaction (PCR) was performed in 15 μL reactions consisting of 7.5 μL 2 × Taq PCR Master Mix, 2 μL primer mix, 1 μL 20 ng DNA template, and 4.5 μL ddH_2_O. PCR amplifications involved an initial denaturation at 96°C for 3 min, followed by 30 cycles of denaturation at 96°C for 30 s, gradient annealing from 62–52°C for 30 s, an extension at 72°C for 1 min, and a final extension at 72°C for 10 min; samples were stored at 4°C. A 3 μL sample of each PCR product was used to verify the amplified sequence length with a DNA marker (Takara DL2000, Japan) by electrophoresis on a 1.5% agarose gel containing ethidium bromide. PCR products were detected by fluorescence using an ABI 3730xl DNA Analyzer. Raw data were strip-typed using GeneMarker software version 2.2.0.

**Table 1:** Primers information of 7 microsatellite markers of *Bellamya purificata*.

Loci	Repeat motif	Primer sequence (5’-3’)	Size range/bp	Fluorescence labeling (5′)
LX001	ATG (142)	F: ATGCCGATTATTCTGATTCTGG	195–312	HEX
		R: ACGCAAGTTTCATTCATGTATGTC		
LX002	ATG (146)	F: GCTCTGTCCAGCAAGAAACTAG	245–465	FAM
		R: ATAGACATCAGTCCGACAAAGC		
LX003	TC (298)	F: CTCCAAAGACTGTTACTGCTACGA	143–285	TAMRA
		R: CACACAAACTAGGTAAGGGGACAT		
LX004	GATA (328)	F: CCTGCGTCAATTTAAAACCATAG	160–444	HEX
		R: GGGTAGGTAGGTGGGTAAGTGAG		
LX005	CAG (41)	F: TTTGCTGCGTTTACTCGTCCTG	217–259	FAM
		R: CTCGTTCTTGGGCTGGGTGTT		
LX006	CAG (178)	F: CATGACATAACACCCCTACCCTT	104–248	TAMRA
		R: AATTTGGTATCTTGGAATCTGACG		
LX007	CCT (238)	F: ACATAAGTGCTTGCGATAGTGCG	92–194	ROX
		R: ATCCCAGTGTCCTCAACCTAAACG		

### 2.3 Data analysis

Polymorphism parameters of microsatellite loci (e.g., average allele number (*Na*), effective number of allele (*Ne*), observed heterozygosity (*Ho*), expected heterozygosity (*He*), Shannon’s Information Index (*I*), Hardy-Weinberg equilibrium (HWE), Nei’s genetic distance, and principal components analysis (PCA)) were evaluated using GenAlEx v6.5 [13]. Polymorphic information content (*PIC*) was evaluated using Cervus 3.0 [14]. Two-by-two genetic differentiation indices (F-statistics, Fst) and molecular analysis of variance (AMOVA) between populations were performed using Arlequin v3.5. Gene flow (*Nm*) between populations was calculated as *Nm* = 0.25 (1−Fst)/Fst. Evolutionary trees were constructed using the unweighted pair group method with an arithmetic mean (UPGMA) generated by PHYLIP [15]. A test was performed using BOTTLENECK v1.2.02 based on a two-stage mutation model because this best fits the mutation process of microsatellites [16]. Structure v2.3.4 was used to analyze population structure based on Bayesian model calculations. Mantel analysis of genetic (Fst/(1−Fst)) and geographic distances was performed with the R software “vegan” package using location information of each snail population recorded by ArcMap [17].

## 3. Results

### 3.1 Genetic diversity among populations

Polymorphism in SSR loci is presented in Table 2. For the 12 *B*. *purificata* populations, 324 alleles were detected at 7 SSR loci. For all loci, mean *Na* was 46 and mean *Ne* was 18.315; values for LX002 were highest (96 and 53.342, respectively) and LX005 lowest (19 and 8.791, respectively). For seven SSR loci, *I* ranged 2.112–4.240 (mean 2.993). *He* and *Ho* of all SSR loci ranged 0.516–0.800 and 0.790–0.981, respectively. The *PIC* of 7 SSR loci was between 0.772 and 0.981; all were highly polymorphic loci (*PIC* > 0.5), and they fit with HWE (i.e., genotype and gene frequency remain constant) (*P* > 0.05).

**Table 2:** Genetic diversity of 7 microsatellite loci in *Bellamya purificata*.

Loci	*Na*	*Ne*	*I*	*Ho*	*He*	*HWE*	*PIC*
LX001	38	8.803	2.746	0.685	0.886	0.228	0.879
LX002	96	53.342	4.240	0.800	0.981	0.185	0.981
LX003	60	28.656	3.707	0.739	0.965	0.234	0.964
LX004	54	17.576	3.314	0.609	0.943	0.354	0.940
LX005	19	8.791	2.387	0.527	0.886	0.405	0.876
LX006	27	4.755	2.112	0.516	0.790	0.347	0.772
LX007	30	6.280	2.444	0.704	0.841	0.163	0.830
Mean	46	18.315	2.993	0.654	0.899	0.274	0.892

Note: *Na*, average allele number; *Ne*, number of effective alleles; *I*, shannon’s information index; *Ho*, observed heterozygosity; *He*, expected heterozygosity; *HWE*, Hardy-Weinberg equilibrium; *PIC*, polymorphic information content index.

For the 12 populations, average values were 13.667 (*Na*), 2.199 (*Ne*), 0.655 (*Ho*), and 0.832 (*He*) (Table 3); *I* values ranged 1.922–2.463, and averaged 2.199 of the 12 populations. The lowest genetic diversity occurred at PL and the highest at LC. All populations were matched with HWE (*P* > 0.05).

**Table 3:** Genetic diversity among 12 populations of *Bellamya purificata*.

Populations	*N*	*Na*	*Ne*	*I*	*Ho*	*He*	*HWE*
Lingyun (LY)	30	12	8.991	2.068	0.652	0.800	0.170
Guilin (GL)	30	17	11.484	2.363	0.659	0.824	0.188
Longzhou(LZ)	30	13	7.451	2.114	0.648	0.816	0.211
Hezhou (HZ)	30	16	9.863	2.463	0.625	0.886	0.291
Pingle (PL)	30	14	7.804	2.243	0.599	0.843	0.288
Wuzhou (WZ)	30	15	8.537	2.301	0.640	0.853	0.250
Luchuan (LC)	30	11	6.959	2.118	0.711	0.842	0.156
Duan (DA)	30	14	9.180	2.344	0.639	0.877	0.272
Shilong (SL)	30	13	6.795	2.119	0.716	0.826	0.129
Longshui (LS)	30	16	9.762	2.208	0.658	0.805	0.191
Quanzhou (QZ)	30	12	7.068	2.123	0.647	0.825	0.225
Longlin (LL)	30	11	5.242	1.922	0.661	0.790	0.158
Mean	30	13.667	8.261	2.199	0.655	0.832	0.211

Note: *N*, number of effective individuals; *Na*, average allele number; *Ne*, number of effective alleles; *I*, shannon’s information index; *Ho*, observed heterozygosity; *He*, expected heterozygosity; *HWE*, Hardy-Weinberg equilibrium.

### 3.2 Genetic structure and differentiation

Over the 12 populations *Fst* values ranged from 0.021 to 0.081, with the lowest genetic differentiation at GL and LY (0.021) (Table 4). *N*m between the 12 populations exceeded 1.0 (ranging from 3.084 to 11.778). AMOVA revealed low levels of genetic divergence among populations (*Fst* = 0.048, *P* < 0.001), with genetic diversity greater within (95.2%) than among populations (4.8%) (Table 5).

**Table 4:** Genetic differentiation index (below diagonal) and gene flow index (above diagonal) of 12 populations of *Bellamya purificata*.

Populations	LY	GL	LZ	HZ	PL	WZ	LC	DA	SL	LS	QZ	LL
LY		11.778	4.785	4.691	4.437	4.342	4.733	5.024	4.478	7.965	5.195	2.841
GL	0.021		7.364	5.722	5.262	5.384	5.891	5.996	6.368	11.112	7.132	3.211
LZ	0.050	0.033		8.952	8.521	9.964	5.389	5.367	7.174	6.975	6.507	6.170
HZ	0.051	0.042	0.027		10.208	9.232	7.078	7.813	6.557	5.312	7.728	6.080
PL	0.053	0.045	0.029	0.024		9.366	5.358	5.799	5.837	5.214	5.850	5.540
WZ	0.054	0.044	0.024	0.026	0.026		8.096	6.831	5.692	5.289	5.981	7.866
LC	0.050	0.041	0.044	0.034	0.045	0.030		6.717	5.305	4.796	6.057	3.777
DA	0.047	0.040	0.045	0.031	0.041	0.035	0.036		7.053	5.568	5.541	4.543
SL	0.053	0.038	0.034	0.037	0.041	0.042	0.045	0.034		6.077	6.129	3.908
LS	0.030	0.022	0.035	0.045	0.046	0.045	0.050	0.043	0.040		8.314	3.084
QZ	0.046	0.034	0.037	0.031	0.041	0.040	0.040	0.043	0.039	0.029		3.683
LL	0.081	0.072	0.039	0.039	0.043	0.031	0.062	0.052	0.060	0.075	0.064	

**Table 5:** AMOVA analysis of 12 populations of *Bellamya purificata*.

Source of variation	Degree freedom	Sum of square	Mean square	Estimates of variances	Percentage variation (%)	F-Statistics	P value
Among Pops	11	135.212	12.292	0.152	0.048	0.048	0.001
Within Pops	644	1939.772	6.060	3.030	0.952	-	-
Total	655	2074.983		3.182	1.000		

**Fig 2:**
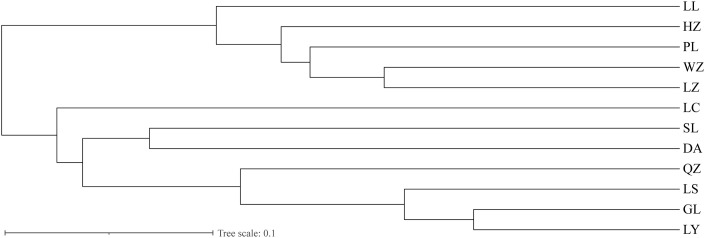
The evolutionary tree of *Bellamya purificata* population was constructed based on Nei’s genetic distance using UPGMA clustering analysis.

**Fig 3:**
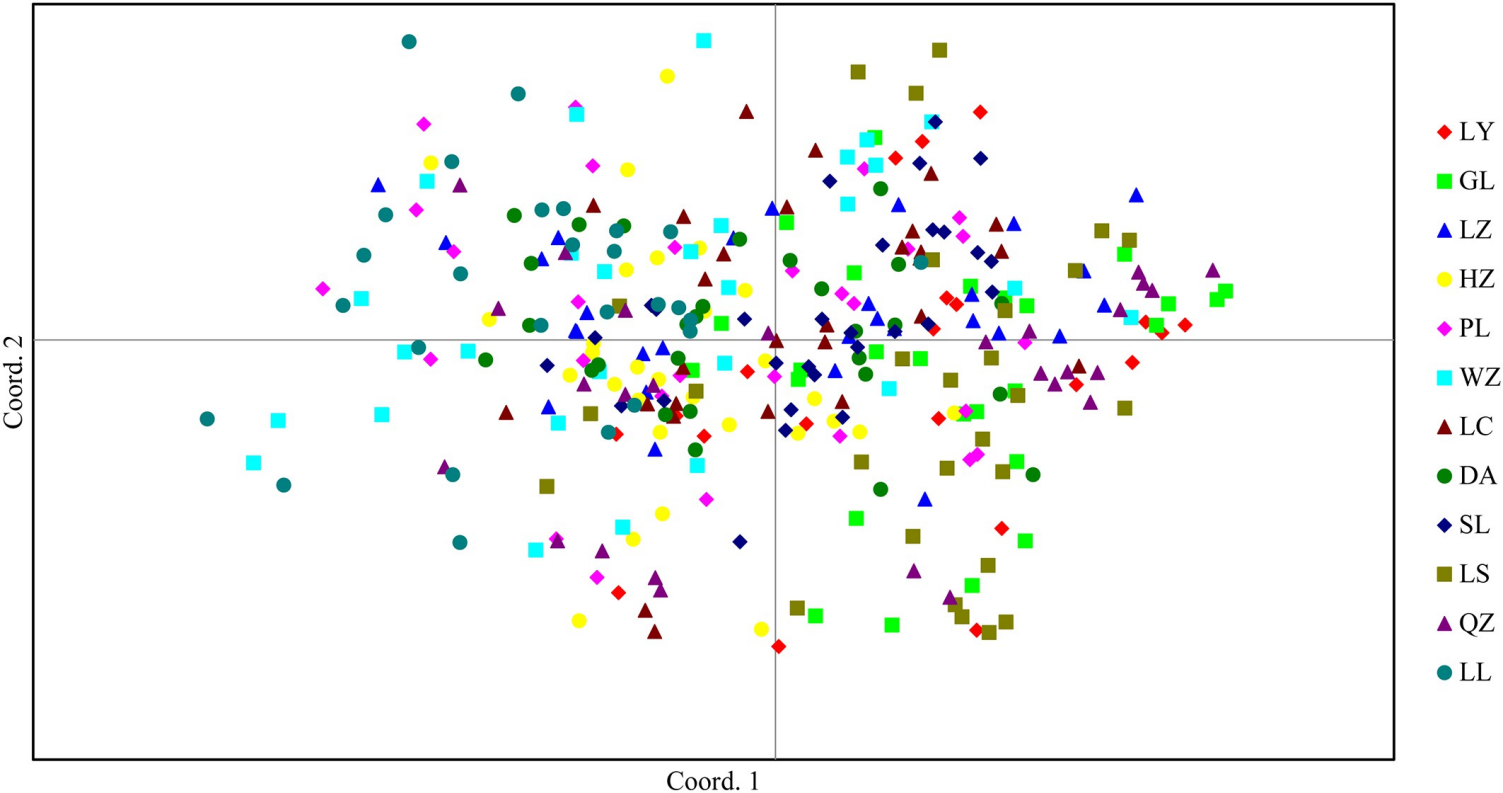
The PCoA analysis of 12 populations of *Bellamya purificata* in Guangxi.

**Fig 4:**
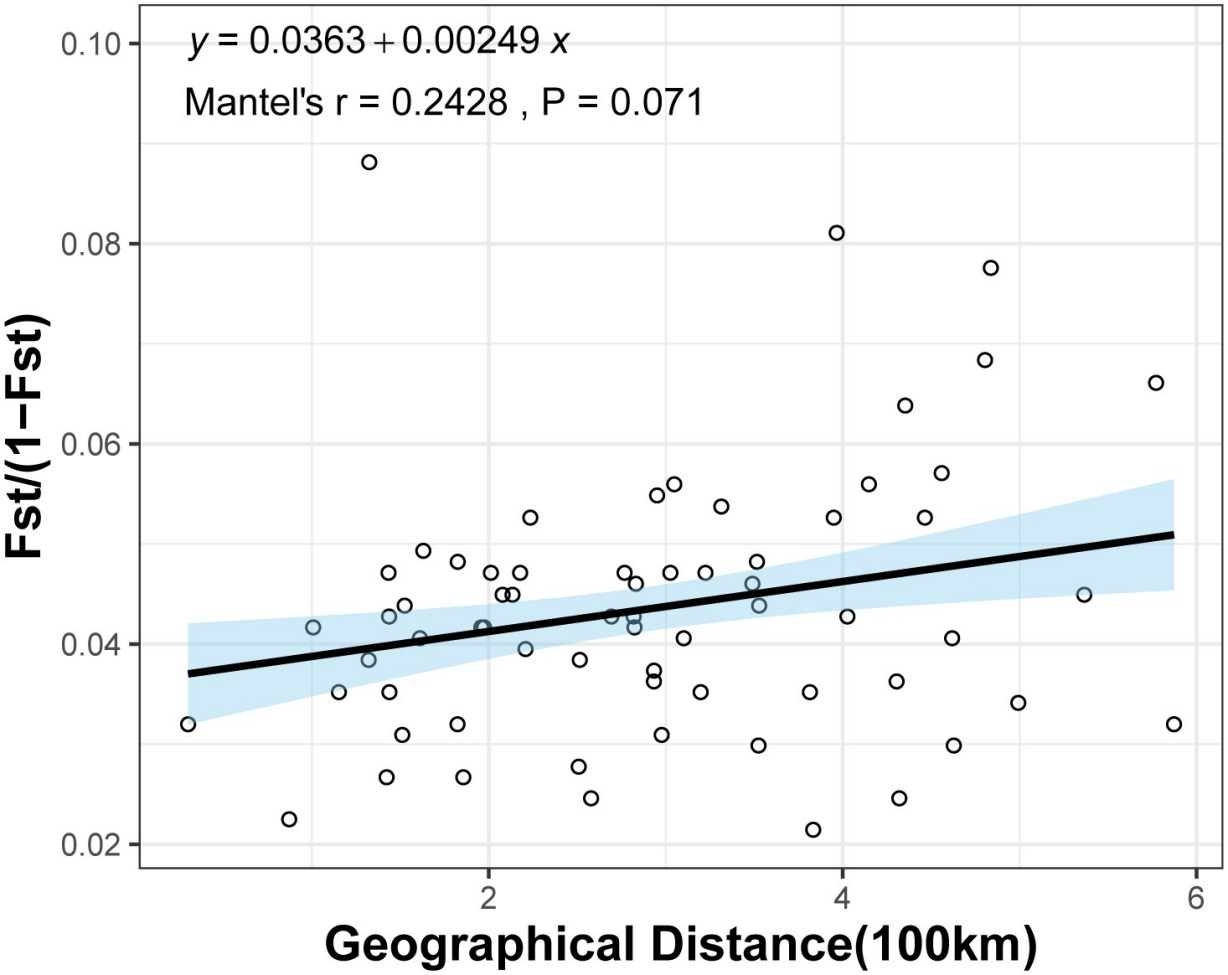
Mantel test between geographical distance and genetic distance.

Genetic distance (*D*) and genetic identity (*DI*) of the 12 populations ranged 0.198–1.129 and 0.323–0.820, respectively (Table 6). Analysis revealed the 12 populations to form two clusters: 1) LL, HZ, PL, WZ, and LZ; and 2) LC, SL, DA, QZ, LS, GL and LY (Fig 2). In general, populations were closely related to each other, consistent with PCA results (Fig 3). Geographical distance had a small effect on genetic distance and no major influence on genetic distance (r = 0.2428, *P* < 0.071) (Fig 4).

**Table 6:** Nei’s genetic distance (below diagonal) and genetic identity (above diagonal) of 12 populations of *Bellamya purificata*.

Populations	LY	GL	LZ	HZ	PL	WZ	LC	DA	SL	LS	QZ	LL
LY		0.820	0.561	0.464	0.488	0.472	0.527	0.522	0.508	0.739	0.576	0.323
GL	0.198		0.700	0.528	0.539	0.546	0.600	0.569	0.634	0.796	0.669	0.365
LZ	0.578	0.357		0.705	0.705	0.753	0.553	0.504	0.676	0.694	0.642	0.665
HZ	0.768	0.638	0.349		0.696	0.645	0.558	0.525	0.555	0.524	0.636	0.599
PL	0.717	0.619	0.350	0.363		0.697	0.501	0.476	0.558	0.558	0.570	0.592
WZ	0.752	0.605	0.284	0.438	0.361		0.651	0.535	0.538	0.563	0.563	0.712
LC	0.641	0.510	0.593	0.584	0.691	0.430		0.550	0.519	0.526	0.579	0.412
DA	0.651	0.563	0.685	0.645	0.742	0.625	0.599		0.600	0.564	0.495	0.464
SL	0.678	0.456	0.392	0.588	0.584	0.620	0.656	0.510		0.634	0.599	0.456
LS	0.302	0.228	0.365	0.647	0.583	0.575	0.642	0.574	0.456		0.731	0.372
QZ	0.552	0.402	0.443	0.453	0.562	0.574	0.547	0.704	0.512	0.314		0.428
LL	1.129	1.007	0.408	0.512	0.524	0.339	0.886	0.767	0.786	0.988	0.850	

### 3.3 Population structure and bottleneck analysis

**Fig 5:**
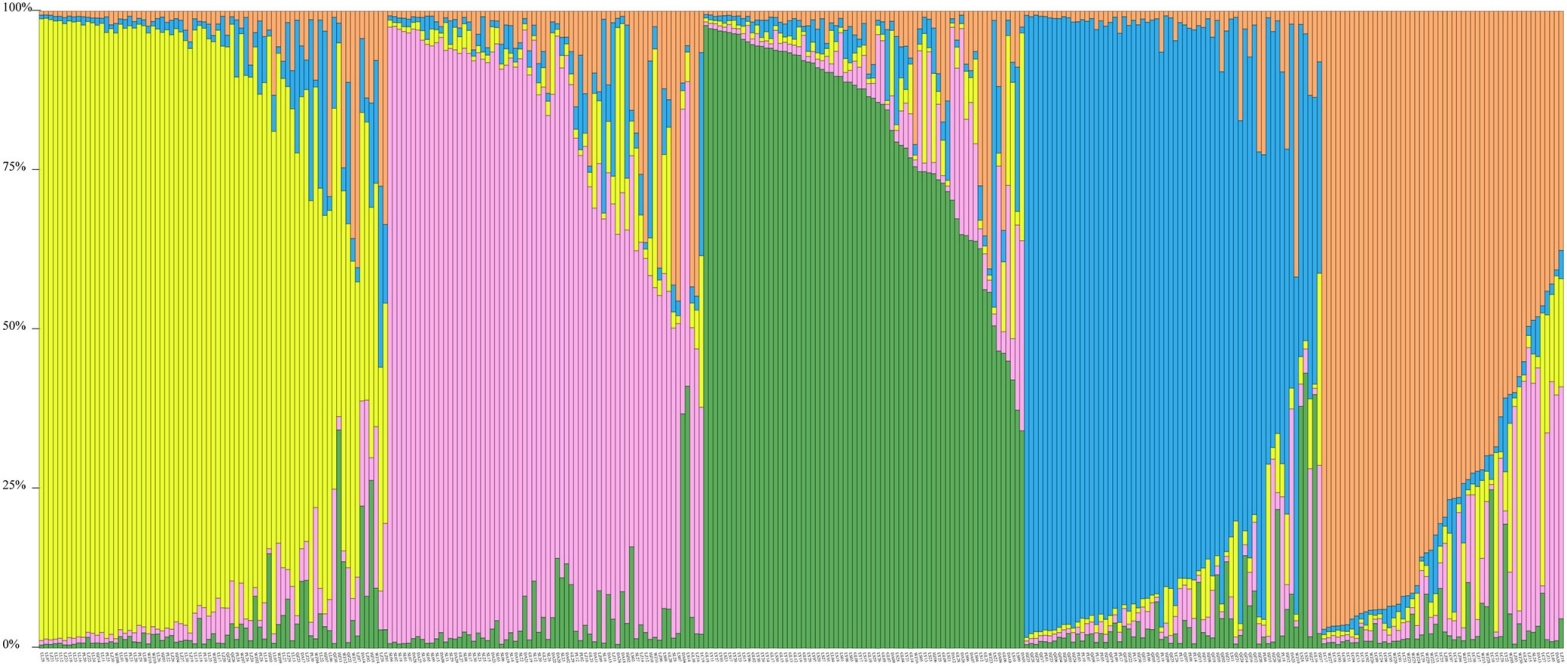
Structural software was used to estimate optimal genetic clustering (K = 5) inference to construct structural bar graphs of *Bellamya purificata*.

Structure-Software produced a maximum ΔK-Value at K = 5. The 360 individuals were divided in five groups (Fig 5), with no population obviously distinguished by this genetic assignment, with most individuals having mixed genetic clusters to varying degrees. No significant heterozygosity-excess was detected in any population using the TPM mutation model (*P* > 0.05), and the normal L-type distribution of allele frequencies indicated that no recent genetic bottlenecks had occurred in any population (Table 7). In addition, there were predicted to be significant heterozygous deficiency (i.e., allelic excesses) in the LS, SL, and WZ populations (*P* < 0.05, under TPM).

**Table 7:** The bottleneck effects of *Bellamya purificata* under TPM mutation model.

Population	Wilcoxon text probability		Mode-shift
	Heterozygosity deficiency	Heterozygosity excess	
DA	0.961	0.055	Nomal L-shape
GL	0.469	0.594	Nomal L-shape
HZ	0.531	0.531	Nomal L-shape
LC	0.531	0.531	Nomal L-shape
LL	0.234	0.813	Nomal L-shape
LS	0.027	0.980	Nomal L-shape
LY	0.344	0.711	Nomal L-shape
LZ	0.469	0.594	Nomal L-shape
PL	0.055	0.961	Nomal L-shape
QZ	0.188	0.852	Nomal L-shape
SL	0.008	0.996	Nomal L-shape
WZ	0.008	0.996	Nomal L-shape

## 4. Discussion

### 4.1 Genetic diversity and differentiation

Genetic diversity is also a product of long-term evolution in species, and the ability of species to adapt to environmental changes increases with it. Compared with that in fish and shrimp, the Conservation, Evaluation, and Development of germplasm-Resources in *B*. *purificata* have received little attention in China. Further research on the genetic diversity of snail populations is necessary to grasp the current status of snail resources in Guangxi, which will provide scientific guidance for the subsequent development, utilization and protection of high-quality resources.

Microsatellite markers are widely used in the genetic analysis of gastropods because of their high polymorphism, ease of operation, and high reliability. Li et al. [18] used 21 microsatellites to genetically analyze selected (3) and wild (6) populations of *Crassostrea gigas*, and found that the genetic diversity of some of the selected populations (LY2-K11) was slightly reduced, which provides valid data for their species selection. Wei et al. [5] successfully developed 22 pairs of polymorphic microsatellite markers based on the full-length transcriptome data of *Cipangopaludina cathayensis*, filling the gap in the lack of available markers for the evaluation of the germplasm resources of this species. Liang et al. [19] used 12 pairs of microsatellites to analyze the *Babylonia areolata* population for three consecutive generations, and found that this population still maintained a high potential for genetic selection. In general, *PIC* is used to evaluate the ability of microsatellite markers to detect polymorphisms in a population [20]. The seven microsatellite loci used in this study showed high levels of polymorphism (*PIC* ranging 0.772–0.981), with the high genetic diversity indices of these markers (*Na* = 46, *Ho* = 0,654, and *He* = 0,899) suggesting that they are useful for studying population differences in this snail species [21].

Generally, the closer the effective allele value (*Na*) is to the absolute value of the average allele number (*Ne*), the more evenly distributed the alleles are within a population. However, *Na*-values exceeding *Ne* occurred in each population, implying that allele numbers were unevenly distributed everywhere, especially at Longshui (16/9.762). A similar phenomenon was reported for the freshwater snail *C*. *cathayensis*, the Japanese seabass *Lateolabrax japonicus* (as *Lateolabrax maculatus*) [22], and the giant river prawn *Macrobrachium rosenbergii* [23]. The Shannon Information Index (*I*) is an important indicator of the evenness of population distribution and the degree of genetic differentiation within a population [24]. Jin et al. [25] reported *I*-values to range 1.723–2.606 using SSR in 11 *B*. *purificata* populations, indicating high levels of genetic diversity. Zhu et al. [26] used eight pairs of microsatellite-primers to analyze the artificially selective populations of *Pinctada fucata* (as *P*. *martensi*), the I-values of which (1.234–1.275) indicate high genetic diversity and large genetic selection potential between them. Our *I-Index* for *B*. *purificata* averaged 2.199 and was highest at Hezhou (2.463) and lowest at Longlin (1.922), indicating a high adaptive potential and genetic capacity in Guangxi [27].

Because genetic heterozygosity is not easily affected by sample size, it better reflects levels of population genetic diversity [28]. The work report an average expected heterozygosity (*He*) for the 12 populations of 0,832, with the lower polymorphism (0.790) of the Longlin population more consistent with results obtained from *I*-value-analysis. The study also report observed heterozygosity (*Ho*) to be lower than the *He*-value and speculate that these populations showed heterozygote deletion (indicating that there were more pure congeners in the population). Dieses may be related to Inbreeding, caused by a combination of *B*. *purificata* habits and the slowing of flowrates following Damconstruction [29, 30].

Calculating genetic distances between populations (based on the Nei-method) is important to study the origin of varieties, analyze relationships between populations, map phylogenetic trees, and manage parental selection [31]. Genetic distance (*D*) and genetic identity (*DI*) values of these 12 *B*. *purificata* populations ranged from 0,198 to 1,129 and 0,323 to 0,820, respectively. Among populations, those at Longlin and Lingyun each had the largest *D* (1.129) and smallest *DI* (0.323), indicating that divergence occurred a long time ago, that these two populations were only distantly related, and that genetic variation was considerable. UPGMA-Clustering produced the same results for these 12 populations.

The coefficient of genetic differentiation (*Fst*) indicates the degree of genetic differentiation in a population [32, 33]. The study *Fst* values ranged 0.021–0.081 (*Fst* < 0.05) and *N*m values ranged 2.841–11.778 (*N*m > 1.0), indicating little genetic differentiation among populations and frequent gene exchange (which can effectively suppress an effect of genetic drift). And this differentiation mainly occurred within the population (95.2%), which was confirmed by the results of PCA analysis. Similarly, the *Fst* value of *B*. *purificata* in the Yangtze River basin was only 0.052, and the main genetic variation (94.84%) came from within the population, which is consistent with the results of this study [10].

### 4.2 Genetic bottlenecks and structure

Genetic Bottlenecks tend to occur in specific populations with diminished adaptive capacity, low genetic variation, and reduced habitat [16]. Although the fishery resources of snails have continued to decline in recent years, no genetic bottlenecks were found in populations of *B*. *purificata*. The study 360 samples from 12 populations clustered in five subpopulations (K = 5), with each sample containing two or more pedigrees, suggesting interbreeding between two or more ancestral subpopulations [34]. Although urbanization, development, river engineering, fishing, and the invasion of alien species have contributed to declines in fish diversity in Guangxi, the *B*. *purificata* population retains high genetic diversity, has low genetic differentiation among populations, is little influenced by geographic distance (r = 0.2428, *P* = 0.071), and gene exchange is evidently frequent (*N*m > 1). The rice-snail model is growing rapidly in Guangxi, which accounts for more than 95% of the total area of rice-snail culture in China. It may be that snails in this province include farm escapees, and that increased flooding in the Xiangjiang and Xijiang river basins facilitates the dispersal of juvenile *B*. *purificata*, contributing to gene flow between populations. Because juveniles adhere to leaves, plastic debris and other floating surfaces, thereby accelerating the efficiency of snail migration in natural basins.

In additional, the study results indicate that there may be significant heterozygosity deficiency and higher *N*e in the LS, SL, and WZ populations, suggesting that this population is presumably associated with infiltration from other populations or has recently undergone population expansion. These findings are consistent with the high levels of genetic diversity described above. Notably, this may reflect the current state of differentiation of all populations and the lack of time for loss of genetic diversity following the census decline. However, *N*e values may have been varying and fluctuating in different parts of Guangxi due to different ecological characteristics, mating system dynamics, gene flow, and genetic mixing [35].

## 5. Conclusion

In the study, the seven SSR-Loci used were highly polymorphic and can be applied to the genetic study and germplasm-resource assessment of *B*. *purificata*. The 12 *B*. *purificata* populations surveyed in the Pearl River system of Guangxi have high genetic diversity, low levels of genetic variation and genetic differentiation, and good status of germplasm resources, with greater potential for exploitation. But with the continuous exploitation and utilization of fishery resources and the gradual expansion of the impact of artificial intervention, the wild snail resources show a trend of gradual decline. So it is necessary to expand the scope of the wild snail and conduct more in-depth research in order to provide more reliable scientific guidance for the conservation and utilization of the snail resources.

## Supporting information

S1 Table Latitude and longitude data used to produce the map of *Bellamya purificata* sampling sites. (XLS)

S2 Table Raw data used for genetic diversity analysis of seven pairs of microsatellite markers. (XLS)

S3 Table Raw data for diversity analysis of *Bellamya purificata* populations using GenAlEx v6.5 software. (XLS)

S4 Table Raw data used for UPGMA clustering analysis of *Bellamya purificata* populations. (XLS)

S5 Table Raw data used for PCoA analyses of *Bellamya purificata* populationsxls. (XLS)

S6 Table Raw data used for the mantel analysis of *Bellamya purificata* populations. (XLS)

S1 File Raw data used for bottleneck analysis of *Bellamya purificata* populations. (PDF)

S1 Video Based on the original results obtained by the structure analysis software. (RAR)
