# Supplementary material for: Genetic diversity and population structure of Bellamya purificata in Guangxi
Source: PLoS One. 2024 Jun 25;19(6):e0305197. doi: 10.1371/journal.pone.0305197 (PMC11198789; doi:10.1371/journal.pone.0305197)
Supplement: S1 File — (PDF) [file pone.0305197.s007.pdf]

```
Title line:""
LX001
LX002
LX003
LX004
LX005
LX006
LX007
Pop
    DA, 0000 0322 2952 1818 0708 0707 0304
    DA, 0000 0332 0252 1643 0909 0909 0314
    DA, 0000 0000 0000 1818 0511 0909 1422
    DA, 0809 1676 1852 2232 0511 0910 0314
    DA, 0000 0000 0000 1841 1010 1015 1421
    DA, 0509 3676 4242 0917 0505 0000 0218
    DA, 1212 4877 1818 1831 0711 0209 1420
    DA, 0516 1251 0000 1616 1018 1010 1316
    DA, 1414 0000 1717 2232 0505 0709 1212
    DA, 0909 4551 0000 0731 0410 0215 0318
    DA, 0808 1619 0000 1732 1111 0209 0318
    DA, 0909 3876 2121 1832 0505 0910 0322
    DA, 0512 0000 0000 1717 1111 0909 1818
    DA, 1432 1516 2626 1843 0508 1016 0414
    DA, 0505 3245 3131 0932 1111 0202 1818
    DA, 0513 4045 0203 1732 0808 0909 0303
    DA, 0000 1922 1744 0552 0412 1212 1422
    DA, 1430 2228 0220 1414 1010 0225 1418
    DA, 0808 5177 0000 2140 0808 0000 1517
    DA, 0808 2862 1818 0330 0209 1010 1616
    DA, 0916 1948 0213 2828 0808 0910 1418
    DA, 1919 2236 0000 2832 1011 0910 1521
    DA, 0512 1640 1856 2128 0808 0215 1717
    DA, 0610 3245 1111 0721 0208 0227 1517
    DA, 0404 1616 2944 1717 0207 0210 1417
    DA, 0909 4876 5054 1616 0215 1010 2121
    DA, 0808 4051 0000 2828 1010 0910 0318
    DA, 0808 1951 0000 2837 0210 0910 1421
    DA, 0000 1640 1856 2837 0208 0210 1414
    DA, 0619 6161 1111 2833 0811 1515 1717
Pop
    GL, 0517 3856 0634 1010 0709 1010 1414
    GL, 1627 3846 1740 0417 0507 1017 0000
    GL, 0808 4158 0606 0000 0000 0000 0000
    GL, 1919 7373 1951 0404 0000 1010 1420
    GL, 0000 3156 0121 1926 0710 1012 0314
    GL, 0321 5359 1939 1842 0711 0910 1416
    GL, 0717 3333 1720 0404 0909 1010 1414
```

GL, 1220 6969 0724 0404 0000 0710 0321
GL, 0000 5561 2231 0407 0909 1217 0323
GL, 0917 2543 1841 0428 0409 1010 0000
GL, 0000 3737 0000 0000 0709 1012 1023
GL, 2024 4373 0000 0426 0913 1010 1218
GL, 1619 2450 3544 0404 0511 1010 0000
GL, 0509 0000 1313 1826 0513 0710 1414
GL, 1921 3644 0000 0404 0709 1010 0926
GL, 0312 1414 3537 1717 0507 1010 1414
GL, 0707 0404 2257 0000 0713 1010 2121
GL, 0920 2831 2731 0404 0505 1010 2121
GL, 0808 1637 0321 1232 0209 1010 1414
GL, 0820 1625 0615 1822 0808 1014 1524
GL, 0319 3241 2126 1825 0909 0925 1212
GL, 0000 4148 3434 0000 0000 0000 1414
GL, 1010 2956 0426 2835 0410 0000 1414
GL, 1010 1843 0000 1212 0808 1012 1314
GL, 0610 5254 5252 1010 0909 1010 1421
GL, 0000 3262 2021 0000 0707 0000 0000
GL, 0808 2236 1518 1825 1010 1010 1328
GL, 0808 0404 2644 1025 0410 1010 1327
GL, 0608 1932 0727 1035 0811 1012 1528
GL, 0619 7070 3547 1235 0808 1012 1415
Pop
HZ, 1926 1111 1919 0718 1212 1015 1515
HZ, 2126 1743 0216 0303 0000 0920 1518
HZ, 1919 1138 1225 2236 0505 0207 0415
HZ, 1019 1620 0000 0707 0810 0209 1020
HZ, 0219 0707 0824 0404 0000 1015 0000
HZ, 1924 1340 1212 0404 0000 1515 0000
HZ, 0619 5757 2230 1010 1112 0212 1516
HZ, 2121 0000 0000 0101 1010 0202 1414
HZ, 2124 1337 0808 0909 0808 0202 1421
HZ, 1919 1720 2137 1818 0811 0912 1416
HZ, 2134 1927 2424 5054 0909 0202 1214
HZ, 0000 2027 2424 0407 0508 0909 1414
HZ, 0725 1119 1634 2236 0507 0707 0414
HZ, 1919 1157 1111 0000 0508 0713 1218
HZ, 0219 1717 0826 0707 0808 1112 1221
HZ, 1922 0000 2231 0000 0816 0000 0000
HZ, 1919 4564 1847 0707 1010 1212 1414
HZ, 1119 4355 0000 0531 0404 0210 0000
HZ, 2226 4646 2626 1717 0811 0423 0219
HZ, 1923 7888 0826 1717 1117 0423 1214
HZ, 1224 2020 2030 0417 0707 1216 1622
HZ, 2126 4555 1945 0417 1919 1212 1212
HZ, 1924 3364 1020 1748 0319 1023 0215

HZ, 0726 2069 2424 1740 0808 0423 0000
HZ, 2222 4545 2323 1717 1111 1023 0212
HZ, 0219 2090 1322 2040 0303 1010 1318
HZ, 1919 1646 1625 1717 1111 0910 0000
HZ, 0234 1673 0808 1015 0719 0410 1519
HZ, 2121 1616 4949 4053 1119 1212 1215
HZ, 0724 1660 2749 2020 0719 1012 1522
Pop
LC, 0610 0000 1838 2734 0409 1012 1414
LC, 0610 0000 1818 1717 0309 1012 0614
LC, 0808 5365 2639 1734 0808 1324 1414
LC, 0819 4380 2639 1717 0417 1024 1414
LC, 0519 6780 2530 1734 1111 1024 0314
LC, 0819 6180 0909 1723 0808 1224 0314
LC, 0819 0000 2638 1723 0409 1224 1415
LC, 0510 2761 1818 1722 0409 1024 1414
LC, 2328 2561 1839 0404 0000 1224 0000
LC, 2828 4369 1039 0404 0507 0000 0000
LC, 0528 2553 3948 1727 0710 1010 0614
LC, 0000 2543 0000 1727 0410 0000 1415
LC, 2834 4361 3948 1717 0911 1012 0614
LC, 0534 2054 3039 1717 1010 0000 1415
LC, 1028 2052 2457 2734 0411 1012 1414
LC, 0505 5461 3739 3434 0410 1024 0622
LC, 2328 2525 0000 1727 0000 1224 1414
LC, 2328 6178 0000 0427 0507 1024 0614
LC, 1010 0000 0000 0909 0711 0914 1516
LC, 0000 0000 0000 2243 0606 0909 0000
LC, 0510 6180 3939 0607 0507 1014 0814
LC, 0000 4365 0000 1617 0707 0909 0325
LC, 0523 6567 1818 1017 0808 0910 1414
LC, 2833 2553 3030 1832 0808 0913 0325
LC, 2833 3553 1839 3136 0518 1224 0218
LC, 2134 3567 2448 0909 0000 1212 1818
LC, 2328 6169 1212 0707 0707 1515 1515
LC, 2133 2742 3737 2232 1111 0910 0618
LC, 2328 6980 1939 1832 0518 0909 0303
LC, 1010 2525 0000 1010 0808 1212 1418
Pop
LL, 0818 3131 2638 0756 0711 1515 0000
LL, 0808 0000 0000 0509 0811 1212 0000
LL, 1919 4081 2638 0127 0812 1212 1415
LL, 0619 4044 0000 1919 0407 1010 0309
LL, 1019 7792 1138 0000 0808 1212 1314
LL, 0810 0909 2039 3852 0511 0000 0304
LL, 0810 3140 2638 0707 1212 1515 1218
LL, 0610 3144 0000 0507 1112 0512 1023

LL,	0819	0000	2638	0749	0404	0202	2022
LL,	0808	1616	2626	0707	1111	0214	0000
LL,	1919	4081	1313	1444	0808	0713	1821
LL,	1919	8183	2638	0606	0912	0202	1416
LL,	1019	0000	0000	0718	1111	1212	0218
LL,	1019	1631	2638	1524	1010	0212	1418
LL,	0808	4040	3847	0000	0808	0213	0215
LL,	0819	3144	0000	0707	0408	0214	0000
LL,	0808	1681	2647	1818	0411	0212	0000
LL,	0819	3181	2638	2932	0505	0707	1421
LL,	0000	0000	2638	5257	0000	1220	0000
LL,	1019	0000	0000	0707	0911	0202	1418
LL,	1919	8181	3838	0000	1212	0203	1014
LL,	0206	0940	0000	4043	0505	0000	1215
LL,	0819	0000	2638	0707	1111	0202	0000
LL,	0819	4081	2638	0707	1111	0915	1421
LL,	1919	3172	0000	0707	0408	0918	1214
LL,	1919	4083	1126	4754	0507	0707	0414
LL,	1019	0000	2026	0707	0909	1015	0000
LL,	1019	1616	2638	0707	1212	0202	1014
LL,	0610	0000	3838	0000	0507	0000	0323
LL,	0610	4081	3858	0000	0000	1220	1415

Pop

LS,	0914	3838	0000	2828	0810	1010	0000
LS,	0919	0000	2626	1832	1010	1212	0000
LS,	0909	3896	0536	0000	0911	1212	2727
LS,	0114	3243	2626	0610	0909	1010	1515
LS,	2137	3973	2734	1125	0909	1012	1822
LS,	1019	3466	3551	1818	0909	0710	1415
LS,	0819	9193	0000	1232	0308	0910	1414
LS,	0819	1946	0000	0535	0911	0210	1414
LS,	0919	6289	2424	1831	0811	1010	0114
LS,	0819	4444	0000	1823	0909	0210	1414
LS,	0000	4667	5054	0000	0810	0202	0214
LS,	0000	3650	1848	1010	0909	1012	1415
LS,	1919	0000	2347	0000	0000	0000	0404
LS,	1919	6262	3152	0000	0000	1515	0303
LS,	1217	4695	1217	1011	0000	1012	0000
LS,	0000	0000	0000	1025	0808	1010	0414
LS,	0303	0404	1729	3132	0510	1010	1527
LS,	0909	4687	1426	1010	1010	1012	1415
LS,	0914	1028	4452	2326	0408	1010	1515
LS,	1414	2794	2960	1023	0409	1010	0000
LS,	1221	6565	1223	0423	0507	1012	1822
LS,	0312	1967	0000	2326	0507	1010	1415
LS,	0912	3158	3234	1023	1016	1010	1421
LS,	1919	3845	3844	1023	0816	1010	0000

LS, 0309 4949 2121 1010 1017 1010 0000
LS, 0000 1738 0000 1023 0809 1010 1415
LS, 0000 2195 2351 1023 0413 1010 0000
LS, 0000 3535 0000 1010 0916 1010 1422
LS, 0909 6870 0431 2323 1016 1010 0000
LS, 0909 4346 2953 1623 0410 1010 0000
Pop
LY, 0812 4953 2727 0404 0000 1015 0000
LY, 0000 4046 0000 0404 0507 1515 1518
LY, 0920 0577 3453 0404 0000 1010 1520
LY, 1425 0000 0505 0000 0913 1010 0000
LY, 0000 3232 0000 0000 0000 1015 0000
LY, 0709 3143 2135 2828 1313 1010 1414
LY, 0912 2877 4954 0000 0507 1215 0000
LY, 0606 0000 0000 0442 0507 1010 1616
LY, 0000 3838 2626 0000 0909 0000 0000
LY, 1621 2424 4646 0000 0507 0000 0314
LY, 1212 0528 3444 1028 0513 1010 0000
LY, 0909 2865 3241 1028 0000 0000 0000
LY, 0915 3140 2248 0728 0507 0000 1515
LY, 0309 4446 1848 0000 1313 0000 1516
LY, 0606 2877 0000 0417 0707 0000 0000
LY, 0909 4848 1526 0000 0507 0000 0000
LY, 0819 0000 0000 0404 0909 1015 1423
LY, 0000 0000 0000 0423 0507 1414 0323
LY, 0919 5863 1832 1028 0513 1010 1014
LY, 1212 4040 4141 4242 0709 1012 1423
LY, 0819 3445 0000 1719 0909 1015 1423
LY, 0606 5381 0000 1026 0708 1012 1414
LY, 0312 3474 1720 2626 0913 1010 1421
LY, 0109 3538 2354 0417 0507 1015 1423
LY, 1619 3442 3950 0000 0507 1017 0312
LY, 0912 5193 3131 2828 1313 0210 1414
LY, 1517 3838 2929 1032 0713 1010 1414
LY, 0000 0000 0000 4245 0713 1010 1414
LY, 1921 3852 2235 1026 0505 1010 1414
LY, 0000 5656 1726 4242 0707 1215 2323
Pop
LZ, 1010 1180 0819 1018 0508 1012 2228
LZ, 0810 3184 1924 0539 0810 0910 1427
LZ, 0610 7578 1325 1235 0808 1212 1429
LZ, 0610 5378 1925 1235 0707 1010 1428
LZ, 0819 2023 2626 0000 0000 0000 1414
LZ, 0819 0973 2630 1825 0808 1012 1414
LZ, 1919 0909 2626 2222 1111 1010 1424
LZ, 0819 3678 2626 1035 0707 1212 1414
LZ, 0819 7680 1626 1832 0909 1025 1222

LZ, 0208 0942 0121 1025 0809 1012 1414
LZ, 1919 2640 1621 0923 0811 1010 1422
LZ, 1919 1273 1313 3539 0909 1010 1414
LZ, 0208 2023 1925 1235 0909 1010 1314
LZ, 0000 1020 1313 2525 0808 1012 1415
LZ, 1919 7780 1330 1021 0909 1010 1429
LZ, 1919 0975 2630 1832 0808 1010 1421
LZ, 1019 8086 1926 1717 0909 1010 1524
LZ, 1919 0909 1326 1010 0809 1010 0000
LZ, 1919 7986 1319 0714 0412 0210 1515
LZ, 0219 2385 1624 1414 0808 0220 1516
LZ, 1919 4040 1319 0707 0404 0202 1419
LZ, 0000 2340 1326 4647 0505 0713 0415
LZ, 0219 2080 1319 0556 0404 0202 0414
LZ, 1019 2075 1925 0707 1112 1012 1518
LZ, 1919 2082 1921 1729 0505 0707 0307
LZ, 1919 2036 1926 0114 0909 0202 1823
LZ, 0210 8084 1638 0707 0911 1020 1215
LZ, 0610 7382 2426 0552 1717 1212 1821
LZ, 0000 2385 1616 0407 0710 0223 1518
LZ, 0819 3680 1325 0707 0909 0202 1521

Pop

PL, 0000 1920 4848 0808 1919 1010 1215
PL, 0000 4560 4751 2121 1818 1212 1530
PL, 3434 3345 2344 5353 0717 1011 0318
PL, 1919 0707 4352 1727 1117 0410 0614
PL, 1619 0808 5059 1717 1717 1010 1415
PL, 1919 0608 2051 1010 1719 1012 1414
PL, 0619 0707 3649 0117 2020 1010 0305
PL, 1919 0856 3250 1717 0811 0410 0000
PL, 0419 0508 0000 1740 1717 1616 1116
PL, 0619 0707 0000 2020 0408 1010 1515
PL, 0606 6767 0000 1517 1117 0910 1215
PL, 1619 2525 0000 1637 1111 1010 1214
PL, 0610 0845 1548 2021 0719 1223 0217
PL, 1019 0808 2547 1720 0111 0410 1219
PL, 0000 0809 3254 0101 1119 1919 0000
PL, 0000 1833 3648 1527 1919 1010 1215
PL, 0619 0707 0000 1717 0419 0910 1214
PL, 1919 0909 0303 2040 0707 1012 1415
PL, 1010 0101 0000 0707 0808 0101 1417
PL, 0000 0822 0000 0707 0404 0202 1415
PL, 0000 0000 0000 5555 0000 0707 1421
PL, 0610 0909 5057 0607 1014 0707 1518
PL, 0819 1135 5050 0707 0411 0202 1521
PL, 0808 0608 0000 0610 1111 0202 0000
PL, 0619 0909 4955 0707 1112 0214 1418

PL, 0619 0207 1728 0707 0404 0202 1415
PL, 0000 0809 0000 0407 1017 0000 1521
PL, 0810 0809 2344 0710 0404 0202 1414
PL, 1919 0811 1111 0607 0909 1515 1517
PL, 0000 3757 1544 0707 1217 0210 0000

Pop

QZ, 0000 0312 0000 1028 0810 1010 1415
QZ, 2338 6767 1230 2326 0817 1010 1415
QZ, 0707 0717 1015 2323 0917 1012 1421
QZ, 0311 1745 2330 2323 0417 1010 0000
QZ, 0000 1945 2222 1010 1011 1212 1415
QZ, 0202 2745 1545 2323 0808 1010 1414
QZ, 0811 0745 0123 1026 0808 1010 1414
QZ, 0000 0000 0000 0923 0811 1010 1522
QZ, 0822 2045 2222 2326 1011 1010 1422
QZ, 0721 0714 2024 1028 0810 1010 1422
QZ, 1122 2045 2238 1623 0811 1010 1422
QZ, 1935 6767 4751 1026 1011 1010 0000
QZ, 0323 0746 2222 1023 1116 1010 1422
QZ, 0321 1445 2638 0926 0916 1010 1414
QZ, 0711 2050 2245 0923 0909 1010 1414
QZ, 1923 2036 2345 1028 0408 1010 1422
QZ, 0221 1745 1545 2323 0811 1010 1414
QZ, 1919 1719 2437 2328 0408 1212 2222
QZ, 0222 0720 2020 1717 0505 0000 0000
QZ, 0722 3389 2353 4354 0811 0215 1527
QZ, 1921 1720 1523 0707 0808 1112 0000
QZ, 0221 2025 1530 0606 1112 0914 1318
QZ, 0303 0000 0000 0707 0404 1214 0219
QZ, 1121 1717 2222 3640 0510 2424 0000
QZ, 0000 1420 2424 0101 0808 1414 0000
QZ, 0819 1962 2638 1111 0404 0214 1218
QZ, 0000 0000 0000 2944 0508 2929 0000
QZ, 0307 2045 1010 0109 1111 1212 0000
QZ, 0000 4444 1247 0551 0808 1414 1524
QZ, 1119 1919 1519 0707 0208 0914 1415

Pop

SL, 0210 2971 2151 3737 0202 1012 1414
SL, 1313 4256 2021 0216 0810 0910 1414
SL, 1019 2839 2128 3235 0000 1027 1515
SL, 0000 4771 1621 2828 0811 0915 1818
SL, 0819 4771 0224 2121 0202 0410 0000
SL, 0808 1932 2121 1717 0808 1212 1717
SL, 0819 2932 2021 1637 1111 0910 1414
SL, 1919 4990 2126 2128 0811 1515 1515
SL, 1019 2947 1616 2828 0810 0810 1919
SL, 1019 1656 0621 0928 1011 1015 1418

SL, 0000 6990 2151 1628 0212 0909 1818
SL, 1016 5154 2951 2837 0210 1015 0305
SL, 1013 1919 0000 1628 0410 1015 1414
SL, 0000 6973 2147 1616 0208 1019 1414
SL, 2931 4247 2147 1616 0408 1015 0614
SL, 1313 6771 2147 2845 1111 1011 0202
SL, 1319 0000 2126 3030 0508 1227 1515
SL, 1319 4790 2126 3235 0808 1027 1515
SL, 1010 0000 0000 1218 0809 0000 2227
SL, 1013 5688 2138 2335 0809 1012 0315
SL, 0819 0000 0000 1016 0810 1010 2828
SL, 0819 4256 0000 1825 0909 1012 1518
SL, 0819 3590 0621 1010 0809 1012 1518
SL, 1919 2961 0000 0539 0917 1010 1515
SL, 1019 4290 2247 1832 1010 0910 1212
SL, 1019 2956 0000 0518 0808 1010 1418
SL, 1010 0000 2038 3239 0404 0910 1228
SL, 0610 0000 2138 2835 0808 1224 2222
SL, 1021 4242 0221 1625 0708 0410 1428
SL, 1313 4788 0000 1832 0809 1012 1429

Pop

WZ, 0819 0000 0000 0707 0909 0221 0000
WZ, 0808 4363 1919 0723 0404 0102 1419
WZ, 0619 7784 2626 0713 1111 0215 0315
WZ, 1919 2341 1318 0728 0808 0202 0000
WZ, 1019 5360 2048 0718 0909 2628 1415
WZ, 0810 3838 0000 0717 0000 0209 0000
WZ, 0610 4059 2133 1018 0405 0101 0000
WZ, 0610 2036 3348 0707 1212 0202 1518
WZ, 0516 8080 0000 0728 0404 0202 1518
WZ, 0819 5970 4055 0707 0404 0202 0000
WZ, 2123 5184 1616 0728 0808 0115 1316
WZ, 1919 5353 0000 1043 0711 0202 1420
WZ, 2336 2541 1826 0507 1717 1115 0000
WZ, 1919 2833 0000 0510 1111 0209 0000
WZ, 2328 3693 0000 0518 1111 0622 0000
WZ, 0923 4653 3131 0000 0000 0606 2227
WZ, 1919 0000 1212 2424 1111 2222 0314
WZ, 0808 2259 2637 0707 1111 0202 0000
WZ, 0719 3333 0000 1717 0407 1012 0614
WZ, 0819 3041 0000 1717 0911 1012 0614
WZ, 0000 0000 2626 1733 0408 1010 0618
WZ, 1019 3389 0426 1727 0408 2424 1415
WZ, 1717 5375 2727 1733 0909 1010 1418
WZ, 0610 4181 0000 1733 0909 1012 1418
WZ, 2836 7879 3135 1727 0409 1324 0322
WZ, 0819 7778 3333 2734 0909 1224 1414

WZ, 2328 6068 2626 2227 0409 1224 0314
WZ, 1919 2938 1111 1727 0911 1010 0614
WZ, 0808 5368 3237 0000 0909 0000 1414
WZ, 1019 3153 0000 0000 0404 1212 1414
